# Comparison of the geographical distribution of feline immunodeficiency virus and feline leukemia virus infections in the United States of America (2000–2011)

**DOI:** 10.1186/1746-6148-9-2

**Published:** 2013-01-05

**Authors:** Bimal K Chhetri, Olaf Berke, David L Pearl, Dorothee Bienzle

**Affiliations:** 1 Department of Population Medicine, Ontario Veterinary College, University of Guelph, Guelph, ON, Canada; 2 Department of Mathematics and Statistics, University of Guelph, Guelph, ON, Canada; 3 Institute of Biometry, Epidemiology and Information Processing, University of Veterinary Medicine Hanover (Foundation), Hanover, Germany; 4 Department of Pathobiology, Ontario Veterinary College, University of Guelph, Guelph, ON, Canada

**Keywords:** Cat, Epidemiology, Retrovirus, Spatial analysis

## Abstract

**Background:**

Although feline immunodeficiency virus (FIV) and feline leukemia virus (FeLV) have similar risk factors and control measures, infection rates have been speculated to vary in geographic distribution over North America. Since both infections are endemic in North America, it was assumed as a working hypothesis that their geographic distributions were similar. Hence, the purpose of this exploratory analysis was to investigate the comparative geographical distribution of both viral infections. Counts of FIV (n=17,108) and FeLV (n=30,017) positive serology results (FIV antibody and FeLV ELISA) were obtained for 48 contiguous states and District of Columbia of the United States of America (US) from the IDEXX Laboratories website. The proportional morbidity ratio of FIV to FeLV infection was estimated for each administrative region and its geographic distribution pattern was visualized by a choropleth map. Statistical evidence of an excess in the proportional morbidity ratio from unity was assessed using the spatial scan test under the normal probability model.

**Results:**

This study revealed distinct spatial distribution patterns in the proportional morbidity ratio suggesting the presence of one or more relevant and geographically varying risk factors. The disease map indicates that there is a higher prevalence of FIV infections in the southern and eastern US compared to FeLV. In contrast, FeLV infections were observed to be more frequent in the western US compared to FIV. The respective excess in proportional morbidity ratio was significant with respect to the spatial scan test (p < 0.05).

**Conclusions:**

The observed variability in the geographical distribution of the proportional morbidity ratio of FIV to FeLV may be related to the presence of an additional or unique, but yet unknown, spatial risk factor. Putative factors may be geographic variations in specific virus strains and rate of vaccination. Knowledge of these factors and the geographical distributions of these infections can inform recommendations for testing, management and prevention. However, further studies are required to investigate the potential association of these factors with FIV and FeLV.

## Background

Infections with feline immunodeficiency virus (FIV) and feline leukemia virus (FeLV) are common and important conditions in cats [1]. Both FIV and FeLV are immunosuppressive retroviruses and associated with a wide array of disease conditions affecting multiple organ systems and susceptibility to opportunistic infections. The most important mode of transmission of both retroviruses is through bites, although other less common modes of transmission such as nursing, mutual grooming or sharing dishes for FeLV [2]; and in utero [3], experimental infection via vaginal mucosa [4], and nursing in neonates [5] for FIV have been reported. Cats at high risk of encountering and fighting with infected cats, and thus getting infected, include those with outdoor lifestyles, and those that are male, adult and non-neutered [6-11].

There is great interest in developing diagnostic tests to identify vaccinated and infected cats and to develop better vaccines to protect uninfected animals [11]. However, little progress has been made in understanding the distribution and causes of FeLV and FIV infections in cat populations. Such knowledge about the prevalence of both infections would assist in defining prophylactic, management and therapeutic measures for stray, feral, and owned cats [12]. Recent studies estimate a sero-prevalence of 2.3% (FeLV) and 2.5% (FIV) in the US [11], and 3.4% (FeLV) and 4.3% (FIV) in Canada [13].

A number of studies suggested that the prevalence of retroviral infections in domestic cat populations may represent regional patterns of infection, which is likely attributable to variable population density, reproductive status, age, gender and housing conditions [14-16]. A study from Vietnam reported FIV sero-prevalence to be higher in the south when compared to the north [17]. Similarly, in Germany, differences in prevalence of FIV between northern and southern states have been reported and attributed to lifestyle, sex and health status of cats [18]. However, regional differences in the US and Canada were still present after adjusting for similar factors [11,13]. Furthermore, even though both infections are known to share similar risk factors, it is unclear whether they also have unique risk factors. Interestingly, in some studies cats tend to have co-infections with both viruses [13,19], whereas in other studies the reverse was shown [20,21]. These contradictory results, and residual variation in sero-prevalence after adjusting for risk factors, might be expressions of geographic variation in the sero-prevalence [11] or unknown spatial factors, which have not yet been explored. Further, geographical variation in the distribution of FIV and FeLV infections has been suggested previously but has not yet been studied using spatial statistics [11,13,22,23].

In this study, we explored the geographical distribution of both viral infections relative to each other in 49 administrative regions (48 contiguous states and the District of Columbia) of the US. If underlying known or unknown risk factors for FIV and FeLV infections vary geographically, then regions with excesses of one infection over the other should exist. The objective of this study was to a) describe the geographical distribution and b) detect high risk areas of FIV and FeLV infections relative to each other.

## Methods

### Description of data

Counts of FIV (n=17,108) and FeLV (n=30,017) positive serological tests (FIV antibody and FeLV ELISA) were obtained for each of the 49 administrative regions of the US from the IDEXX laboratories’ public access website on FIV, FeLV and heartworm infections [24]. The data encompass positive test results for FIV and FeLV from IDEXX sponsored prevalence studies [11,25], IDEXX VetLab Station data reported from veterinary practices, and IDEXX reference laboratories' results collected from 2000 to 2011 [24]. The screening serology for FIV and FeLV entails use of antigen and antibody capture Enzyme-Linked Immunosorbent Assays (ELISA) [26], with sensitivities of 100% and 97.6% and specificities of 99.5% and 99.1%, respectively. The assay tests for both viruses in a combined kit format. Each administrative region was geo-referenced to latitude and longitude coordinates of the respective administrative region centroid. These centroids were extracted from the digital map of the US states, in Environmental System Research Institute (ESRI) shapefile format [27] obtained from the US Census Bureau's 2010 geographic data website [28], using the R statistical software [29].

### Disease mapping - choropleth maps

The proportional morbidity ratio (PMR) of FIV to FeLV infection was estimated for each administrative region and a choropleth disease map was used to visualize the spatial pattern of PMR. Choropleth maps represent regional values such as the prevalence by colour scales where each scale represents a discrete value or a range of values [30]. All maps were displayed in Albers equal area conic projection.

Conventionally, a proportional morbidity/mortality ratio for a particular disease is the observed proportion of illness/death due to a cause over the expected proportion. The expected proportion is the number of illness/death in a reference population from the specific cause over all illness/death in that population [31]. The PMR is likewise defined as the ratio of two morbidity measures, such as the sero-prevalence for two infections:

(1) $${\text{PMR}=p}_{1}{/p}_{2}=\left(m_{1}/n_{1}\right)/\left(m_{2}/n_{2}\right)\text{,}$$

where m_1_ and m_2_ denote the number of cases for FIV and FeLV infections respectively, similarly n_1_ and n_2_ denote the number of tested cats for the respective infections.

For the present study only the total number of cats that tested positive for either infection was available. However, cats are typically tested with a dual ELISA test that is able to detect antibodies to FIV as well as FeLV antigens [32] at the same time. Furthermore, the American Association of Feline Practitioners recommends testing for both infections at the same time [9,33]. Therefore, on the assumption that a combination ELISA was applied to test for both infections simultaneously, the number of tested individuals is the same for both infections (i.e., n_1_ = n_2_). Therefore the PMR formula reduces to PMR = m_1_/m_2_.

Therefore, the PMR _(FIV, FeLV)_ equals the number of cats testing positive for FIV over the number of cats testing positive for FeLV. An area, or administrative region, with PMR >1 represents an excess of FIV infections compared to FeLV infections. Alternatively, a PMR <1 for an area indicates excess of FeLV infections relative to FIV infections in that area. Respective PMRs for each administrative region were visualized as choropleth maps using breaks based on the quintiles of the empirical distribution of the 49 administrative region PMRs.

### Disease cluster detection - spatial scan test

In order to compare the relative distribution of FIV to FeLV (i.e., the PMR), data were aggregated to administrative region centroids. Statistically significant high risk clusters of FIV (or FeLV) infection were identified using a weighted normal spatial scan test [34] as implemented in SaTScan™ [35]. Since the PMR is a continuous variable and its geographical distribution was of interest, the spatial scan test based on the normal probability model was used to detect clusters of high or low PMRs. The normal spatial scan statistic applies to continuously distributed data and not just Gaussian, i.e. normally distributed data [34]. Moreover, the “weighted” version of the normal spatial scan test was used, which allows to adjust for varying regional uncertainty in the PMR estimates, due to varying sample sizes. The weights for each of the 49 administrative regions were estimated as the mean of the total number of cats testing positive for FIV and FeLV infections in each region.

The spatial scan test identifies potential clusters of high or low risk by moving circular windows of varying radius (size) and location (region centroids) across the study area. The one-sided test was performed to identify significant high and low risk clusters. A high risk cluster was defined as an aggregation of administrative regions with mean PMR >1 (i.e. neighbouring regions in which FIV was more frequent), and a low risk cluster for mean PMR <1 (i.e. neighbouring regions in which FeLV was more frequent). The null hypothesis of the one-sided spatial scan test states the mean of the PMR as constant throughout the study area, i.e. not different inside and outside the scanning window [34]. The weighted normal spatial scan statistic therefore identifies as a cluster a group of two or more regions with mean PMR higher or lower than outside the cluster.

The maximum window size was set to 50% of all administrative areas. A p-value was obtained by Monte Carlo hypothesis testing with 999 iterations and the significance level was chosen to be α = 0.05. Areas of relative FIV excess identified by the spatial scan statistic were visualized by highlighting the boundaries of the states included in the most likely cluster on a choropleth map of the PMR of FIV to FeLV infection. The same approach was used to visualize areas of FeLV excess.

## Results

**Figure 1:**
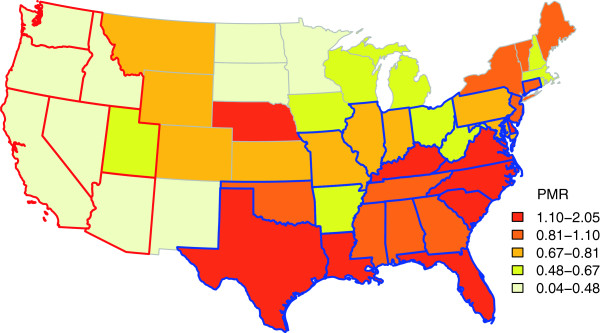
**Choropleth map of proportional morbidity ratios (PMR) of FIV to FeLV infection in the US.** Colors on the map depict the range of PMR values for 48 contiguous states and District of Columbia of the US. Red and blue borders indicate high risk areas of FIV and FeLV infection relative to each other. These high risk areas were identified as 'clusters' by spatial scan test using a weighted normal model. Areas with blue borders depict administrative regions where FIV infections are greater than FeLV among cats. Areas with red borders indicate administrative regions where FeLV infections in cats are greater than FIV.

The descriptive statistics of the data are presented in Table 1. A total of 14/49 administrative regions had a proportional morbidity ratio (PMR) >1 and 35/49 administrative regions had a PMR <1. PMR ranged from 0.04 to 2.05. The FIV and FeLV infections had distinct spatial distribution patterns. The choropleth map revealed more frequent infection with FIV compared to FeLV in the southern and eastern US. In contrast, FeLV infections were observed more frequently in the western and north-central US compared to FIV (Figure 1).

**Table 1:** Descriptive statistics of FIV and FeLV infections, and the proportional morbidity ratios (PMR)

**Parameters**	**Mean**	**Median**	**Range**
Number of FIV Positives	349	92	4 - 4610
Number of FeLV Positives	612	163	3 - 9113
PMR	0.79	0.72	0.04 - 2.05

The spatial scan test detected two high risk clusters. One high risk cluster consisted of administrative regions having an excess of FIV infections (Mean PMR =1.03, p <0.05, 24 administrative regions), and the other high risk cluster consisted of administrative regions having an excess of FeLV infections (Mean PMR = 0.14, p < 0.005, 7 administrative regions) (Table 2 and Figure 1).

**Table 2:** Characteristics of high risk areas (clusters) detected by spatial scan test for FIV and FeLV infections

**Cluster Type**	**Inside cluster**		**Outside cluster**		**Cluster radius (kms)**	**p-value**
	**Number of states**	**Mean PMR**	**Number of states**	**Mean PMR**		
FIV	24	1.03	25	0.35	1688.96	0.02
FeLV	7	0.14	42	0.95	1127.96	0.002

## Discussion

This exploratory analysis identified that areas of relative excess of FIV and FeLV exist in the US. Both the choropleth maps of PMR and the spatial scan test for evidence of high risk clusters identified similar areas of relative excess of one infection over the other. Since it is assumed that both infections share similar risk factors, it would be expected that the occurrence of both infections relative to each other would be more or less uniform throughout the US. However, our spatial analyses show that higher numbers of FIV infections were reported in the southern and eastern US compared to FeLV infections. In contrast, reported FeLV infections were observed to be higher in the western and north-central US compared to FIV infections. These results suggest that the relative excesses of one infection over the other may be the result of different factors affecting these geographical areas. The distinct pattern in the geographical variation of the PMR can be explained in a number of ways relating to the agent, environment and host factors. For example, the dominant viral strain might vary over the study area. Furthermore, vaccination management, level of veterinary care, and thus the age and survival times of cats, may differ from place to place.

Factors that play a role in promoting aggression and bites are known to be most important in the transmission of infection from one cat to another for both FIV and FeLV. These known risk factors include feline population type (pet, stray and feral), cat density, sex, age, neutering status, and access to outdoors [6,7,11]. Previous studies indicated that FeLV infection is age dependent and primarily acquired by “friendly” cats through prolonged close contact between virus shedders and susceptible cats involving mutual grooming, sharing of food and water dishes, and use of common litter areas [36]. However, other studies have indicated adulthood, outdoor lifestyle, neutering status, and fighting to be associated with FeLV as well [11,13,18]. Thus, it is difficult to discern whether these known risk factors, being unique to one infection or the other, could lead to such geographical variability, and results suggest the existence of an unknown spatial risk factor. Further, previous studies have found differences in sero-prevalence across the US despite controlling for these factors [11].

Identification and segregation have been the most important tools in the control of both infections [9]. Although a FIV vaccine was introduced in 2002 in the US, its efficacy remains controversial; whereas vaccination has been attributed as a factor associated with the decreasing prevalence of FeLV [9]. It is possible that the prevalence of vaccination may influence the infection patterns observed in this study. The decision to vaccinate a pet would be dependent on owner compliance and related to their socio-economic status, and these factors would vary geographically.

Previous studies have found that approximately 50% and 80% of FeLV infected cats in multi-cat households are likely to die in the two and three years following diagnosis, respectively [37,38]. On the other hand, clinical signs in most FIV infected cats are reflective of secondary diseases, and FIV is not thought to cause severe clinical illness in naturally infected cats until advanced age. In fact, with proper care FIV infected cats can live for many years [39]. Therefore, one would expect to find more FIV than FeLV survivors when sampling from, on average, older populations. Further, cats testing positive for FeLV are likely to be much younger than those testing for FIV, which also implies that most older cats that are FIV positive are more likely to be pets, and therefore may belong to people of higher socioeconomic status than cats that are young, FeLV positive, and more likely to be owned by shelters or catteries.

Different viral clades or strains of FIV are known to predominate in different geographical regions and could reflect the patterns observed in this study. Although clade-specific information was not available for this study, clade A viruses are common in the western US, whereas clade B viruses predominate in the eastern US [40]. However, the association between viral clades and pathogenicity is unclear [41].

It is important that limitations be considered when interpreting results from this study. The observed variability in infection could be reflective of diagnostic submissions specifically to IDEXX laboratories. This could lead to admission risk bias, a form of selection bias, as is common with registry or hospital based studies, particularly if preference of diagnostic lab by sample submitters in an area is related to the true prevalence of either FIV or FeLV.

Further, sero-prevalence of co-infections with FIV and FeLV ranging from 0.3% to 1.6% have been reported in North American cats [11,13,19,42]. However, estimation of the PMR assumes both the infections to be independent of each other. Not accounting for coinfections would lead to biased estimates of the PMR. However, as the proportion of coinfections increases, the PMR converges to 1; this means the bias is towards the null. Thus, the PMR estimate in this study is rather conservative, i.e. less extreme. Similarly, the result of the spatial scan test is believed to be conservative, i.e. any significant results are truly significant.

The scan test used in this study implements circular shaped windows to detect clusters which may pose a problem when the outcome of interest is aggregated in a non-circular fashion. The scan test may, for example, detect a larger circular high risk cluster by including surrounding regions of low risk [43]. Though other non-circular scan tests have been proposed in the literature, none allow for continuously distributed spatial observations such as PMRs.

For this study, an exploratory approach was applied to compare two similar infections and explore the areas of relative excess rather than derive risk estimates for each area primarily because the underlying population (total number of tested cats in each administrative region) was not known. Such an approach has been reported in the veterinary literature to compare relative excess of one disease to the other [44]. An advantage of these study designs (e.g. case-case study) is that factors may be identified as more important for one disease than the other.

The evidence of distinct clusters of infection necessitates the need to investigate overall spatial dependence in the occurrence of cases (clustering), and if these are identified, to adjust for their presence when evaluating the association of putative risk factors to these infections. Ignoring clustering may result in biased standard errors and thus can compromise risk factor studies [45].

## Conclusions

In this study we have identified geographical patterns in the distribution of the proportional morbidity ratio of FIV to FeLV infection among cats in the 49 administrative regions of the US over the period 2000 to 2011. These patterns might be an expression of geographic variation in the pathogenicity of viral strains that are not evenly distributed in the study area, reflect geographical differences in vaccination practices or relate to differences in survival times after infection. Further studies are warranted to explore the association of these proposed factors with respective infections that allows for adjustment of spatial clustering if present in the data.

## Abbreviations

FIV: Feline Immunodeficiency Virus; FeLV: Feline Leukemia Virus; US: United States of America; ELISA: Enzyme Linked Immunosorbent Assay; PMR: Proportional Morbidity Ratio; ESRI: Environmental Systems Research Institute.

## Competing interests

The authors declare that they have no competing interests.

## Authors’ contributions

BC carried out the data acquisition, statistical analysis and drafted the manuscript. OB conceived of the study, and participated in its data acquisition, analysis and helped to draft the manuscript. DP and DB provided intellectual inputs on study design, analysis and contributed to manuscript revision. All authors read and approved the final manuscript.
